# Data Consistency of Two National Registries in Iran: A Preliminary Assessment to Health Information Exchange

**DOI:** 10.34172/aim.30023

**Published:** 2024-07-01

**Authors:** Mohammad Dashtkoohi, Mohammad Poursalehian, Zahra Azadmanjir, Masoomeh Vaeidi, Mahdi Mohammadzadeh, Mahdi Sharif-Alhoseini, Khatereh Naghdi, Marzieh Moniri Asl, James Harrop, Vafa Rahimi-Movaghar

**Affiliations:** ^1^Sina Trauma and Surgery Research Center, Tehran University of Medical Sciences, Tehran, Iran; ^2^Students’ Scientific Research Center (SSRC), Tehran University of Medical Sciences, Tehran, Iran; ^3^Brain and Spinal Cord Injury Research Center, Neuroscience Institute, Tehran University of Medical Sciences, Tehran, Iran; ^4^Department of Health Information Management, School of Allied Medical Sciences, Tehran University of Medical Sciences, Tehran, Iran; ^5^Trauma Research Center, Kashan University of Medical Sciences, Kashan, Iran; ^6^Health Information Technology Department, Urmia University of Medical Sciences, Urmia, Iran; ^7^Department of Neurological Surgery, Thomas Jefferson University Hospital, Philadelphia, PA, USA

**Keywords:** Electronic health records, Health information exchange, Spinal cord injuries, Trauma centers

## Abstract

**Background:**

The National Spinal Cord Injury Registry of Iran (NSCIR-IR) and the National Trauma Registry of Iran (NTRI) were established to meet the data needs for research and assessing trauma status in Iran. These registries have a group of patients shared by both registries, and it is expected that some identical data will be collected about them. A general question arises whether the spinal cord injury registry can receive part of the common data from the trauma registry and not collect them independently.

**Methods:**

We examined variables captured in both registries based on structure and concept, identified the overlapping period during which both systems recorded data in the same centers and extracted relevant data from both registries. Further, we evaluated the data for any discrepancies in amount or nature and pinpointed the underlying reasons for any inconsistencies.

**Results:**

Out of all the variables in the NSCIR-IR database, 18.6% of variables were similar to the NTRI in terms of concept and structure. Although four hospitals participated in both registries, only two (Sina and Beheshti Hospitals) had common cases. Patient names, prehospital intubation, ambulance arrival time, ICU length of stay, and admission time were consistent across both registries with no differences. Other common data variables had significant discrepancies.

**Conclusion:**

This study highlights the potential for health information exchange (HIE) between NSCIR-IR and NTRI and serves as a starting point for stakeholders and policymakers to understand the differences between the two registries and work toward the successful adoption of HIE.

## Introduction

 In accordance with the directives established by the International Organization for Standardization (ISO), electronic health records (EHRs) are defined as computer-based systems designed to archive data regarding an individual’s medical condition.^1^ The clinical environment has been transformed by EHRs. It has simplified clinical workflows and allows for instant access to patient data.^2,3^ However, it is crucial to acknowledge the sensitive nature of health-related data and related concerns.^4-7^ Data is considered the “fuel” of eHealth by the World Health Organization (WHO) and it is emphasized that universal health coverage requires EHRs.^8^ Management of EHRs can be challenging due to fragmented, duplicated, and inconsistent data. To address these challenges, the concept of health information exchange (HIE) has been developed to facilitate the secure and efficient exchange of EHRs between different health systems.^9^

 HIE plays a critical role in facilitating timely and efficient healthcare delivery, particularly in cases where patients are receiving care from multiple healthcare providers.^9^ The benefits of HIE extend to all parties involved in the healthcare industry, encompassing patients, providers, payers, and policymakers, as evidenced by research.^10-12^ However, HIE can also be challenging. These challenges can have significant implications for patients’ well-being, potentially even leading to life-threatening outcomes.^13^

 Several obstacles come with HIE, such as costly installation expenses, low-quality data, reluctance on the part of patients and health centers due to fears of competition or established procedures at medical facilities, or lack of a uniform format for data transmission. It is crucial to overcome these barriers for HIE to be efficiently implemented.^14,15^

 The National Spinal Cord Injury Registry of Iran (NSCIR-IR) was launched in 2015 to improve the quality of care for patients with spinal cord or spinal cord injuries.^16-18^ Similarly, the National Trauma Registry of Iran (NTRI) was launched in 2016 to address the shortcomings of the Hospital Health Information System (HIS) in assessing the trauma status of patients.^19,20^ According to the inclusion criteria of both registries, patients with traumatic spinal cord injury should be registered in both registries with the expectation that their data would match. The study assessed the success of these registries based on a range of factors including the number of matching patients, the consistency of data items, and the coherence of values for patients who were identified in both systems. Furthermore, the study aimed to identify any reasons for inconsistencies or conflicts in the information recorded between the two registries.

## Materials and Methods

 This study entailed analysis of the structures (format) and concepts of data elements in the registry systems. The data format in this study denotes the predefined method of inputting and storing information in a registry, and a similar concept refers to the recording of identical entity data in two registries regardless of data formats. During the study, four hospitals were collaborating with NSCIR-IR and NTRI. To accomplish this study, we examined the period during which both registries performed case finding and patient registration. We then collected data from the two registries across all four hospitals. To identify common cases in the two registries, we used different identifying factors such as the patient’s first and last name (considering spelling variations), national code, referral number, patient record number, and phone number. In order to evaluate the consistency of data values between common patients in two registers, ten cases were randomly selected and analyzed for inconsistencies by two researchers. Inconsistencies and discrepancies were marked, and an independent third observer created a checklist based on the identified type of conflict in the notes. The same two researchers then used the developed checklist to detect data inconsistencies in all common cases. The types of discrepancies for each item were measured. Ultimately, the technical expert panel at the registry center level, in collaboration with registry leaders, assessed the causes and sources of the discrepancies. The classifications used for data inconsistencies or discrepancies between the two registers are presented in Table 1.

**Table 1:** Type of common variables and potential inconsistencies in each group

	**Type of Common Variable**	
	**Similar Data Format**	**Similar Concept**
Type of inconsistency	Missing data	Missing data
	Typographical errors	Different entered values (data conflict)
	Different entered values (data conflict)	Different levels of details (data granularity)
	Different data entry methods	

Note: Discrepancy or conflict of data with similar structure and concept was classified into the following categories: (i) missing data values (e.g. the field for the province of the accident location was filled in NSCIR-IR, but in NTRI this field was empty and vice versa), (ii) typing errors (e.g. the field for the telephone number is filled in one register as 0912XXX8872 and in the other as 0912XXX8827), (iii) different data values or what we call data conflict (e.g. the cause of an accident is a fall in one database and a car accident in the other), and (iv) different methods of data entry (e.g. blood pressure is 120 in one registry and 12 in the other). We classified inconsistencies in data elements with similar concepts but different structures (data format) into the following classes: (i) unanswered questions, (ii) different data values or, as we call them, data conflicts, (iii) different levels of detail of the data value (so-called data granularity), e.g. the field for comorbid conditions was filled in with diabetes mellitus and hypertension in one database, whereas only diabetes mellitus was entered in the other database.

## Results

 A total of 69 conceptual common variables were identified between the two registries, which was equal to 23.71% of the NSCIR-IR variables. In addition, 54 variables were found that have identical concepts and formats. Further details of the common variables are shown in Table 2. The study period for each center and the number of matched registered cases for each center are shown in Table 3. The results showed no commonality between the entries in our registries during the selected period at the two collaborating hospitals; so, we excluded them from further analysis to avoid overlapping bias in the results. In addition, neither center had a complete record of patients.

**Table 2:** Number of Similar Data Elements (Variables) Between NSCIR-IR and NTRI

	**NSCIR-IR**	**NTRI**	**Variables with Common Concept**	**Variables with Common Format**
Identity elements	20	28	19 (95%) Name, Last name, National ID, Passport ID, Date of birth, Nationality, Education, Record Number, Admission number, Marital status, Province, City, Address, call numbers (4), Gender, and Job.	15 (75%) Name, Last name, National ID, Passport ID, Date of birth, Nationality, Education, Record Number, Admission number, Marital status, Province, City, Address, Phone number of patients, and Phone number of relatives.
Pre-hospital elements	33	50	31 (93.93%) Date and time of injury event, injury cause, safety device, height in falling, activity in the event, county, province, city, place of the injury event, patient transfer, reasons for transfer, method of transport to the hospital, actions descriptions, source of information, inpatient duration, immobilization, EMS times (3), prehospital GCS, cardiac arrest, cardiopulmonary resuscitation, external causes codes.	24 (72.72%) Date and time of injury event, injury cause, safety device, height in falling, activity in the event, county, province, city, place of the injury event, injury cause, patient transfer, reasons for transfer, method of transport to the hospital, actions descriptions, inpatient duration, EMS times (3), prehospital GCS, cardiac arrest, cardiopulmonary resuscitation, external causes codes.
Emergency elements	20	23	15 (75%) Date and time of emergency admission, date, and time of departure from emergency, patient status, blood pressure, pulse rate, respiratory rate, respiratory way status, intubation, O_2_ saturation, GCS (4), comorbid disorders, smoking history.	12 (52.17%) Date and time of emergency admission, date, and time of departure from emergency, blood pressure, pulse rate, respiratory rate, respiratory way status, intubation, O_2_ saturation, GCS (4).
Injury diagnosis elements	11 + 163^1^	4	0 (0%)	0 (0%)
Intervention elements	14	24	1 (0.07%) Surgery performed or not	1 (0.07%) Surgery performed or not
Side effect elements	16 + 10^2^	2	0 (0%)	0 (0%)
discharge elements	4	7	3 (75%) ICU stay, date of discharge, patient discharge status.	2 (50%) ICU stay, date of discharge.
Total	291	104	69 (23.71% of elements of NSCIR were shared with NTRI)	54 (18.55% of data elements were gathered with the same format and concept)

NTRI, National Trauma Registry of Iran; NSCIR-IR, National Spinal Cord Injury Registry of Iran. Note: Percentages are calculated in relation to the elements of NSCIR-IR. ^1^163 variables are related to ASIA items that are not in the NTRI registry ^2^The NSCIR-IR includes 10 items related to complications and 16 items related to bedsores

**Table 3:** Number of Matched Cases in the Joint Centers in NSCIR-IR and NTRI According to Different Identifiers as Key

**Hospital Names**	**Date of Study of Registries**				**The Number of Matched Cases Using the Specified Variables**				
	**Start Date**	**End Date**	**No. of Recorded Patients with Spinal Injury by NTRI**	**No. of Registered Patients by NSCIR-IR**	**Registered Names of Patients (with Consideration of Writing Differences)**	**National ID**	**Admission Number**	**Recording Number**	**Mobile Phone Number**
Sina, Tehran	24 July 2016	19 December 2017	45	65	45	39	41	37	25
Shahid-Beheshti, Kashan	17 July 2017	9 March 2018	47	47	37	31	17	17	35
Imam-Khomeini, Urmia	15 October 2017	19 February 2018	0	50	0	0	0	0	0
Shahid-Rahnamoon, Yazd	23 November 2017	17 April 2018	10	30	0	0	0	0	0

 According to the discussion on the expert panel, the difference in case finding is due to the source of data collection. In contrast to the NTRI, the cases in the NSCIR-IR were obtained from various sources in addition to the list of patients in the emergency department, such as the list of patients admitted to the wards, reports from head nurses and neurosurgery residents, and outpatients who were admitted directly were also recorded.

 The nature and percentage of data inconsistency between the two registries and the details are shown in Tables 4 and 5. The most common discrepancies in demographic data were related to occupations, due to differences in registration methods, and birth dates, especially for immigrants and non-Iranian patients due to the sources used for birth dates.

**Table 4:** Percentage of Data Inconsistencies for Data Elements Between NSCIR-IR and NTRI for Matched Cases

**Data Element**	**Total Inconsistency (%)**	**Missing in NTRI**	**Missing in NSCIR IR**	**Missing in Both**	**Different Data Value**	**Misspelling in NSCIR IR**	**Misspelling in NTRI**	**Different Spelling**	**Different Data Entry**	**Different Details Level**
National ID	4.76	0	0	25	75	0	0	0	0	0
Date of Birth	20.24	23.53	0	0	76.47	0	0	0	0	0
First name	5.95	0	0	0	0	0	0	100	0	0
Last name	10.71	0	0	0	11.11	0	11.11	22.22	0	55.56
Nationality	5.95	0	0	0	60	0	0	0	0	40
Education	11.90	40	0	0	30	0	0	0	30	0
Record Number	19.05	31.25	0	0	68.75	0	0	0	0	0
Admission number	10.71	44.44	0	0	55.56	0	0	0	0	0
Marital status	10.71	55.56	0	0	44.44	0	0	0	0	0
Province, city	11.90	40	0	0	50	0	0	10	0	0
Address	39.29	15.15	0	0	33.33	0	0	9.09	0	42.42
Home phone number	44.05	24.32	2.70	27.03	5.41	0	0	0	0	40.54
Mobile phone number	23.81	20	5	0	75	0	0	0	0	0
Job	39.29	6.06	0	0	21.21	0	0	0	72.73	0
Transfer to hospital	35.71	0	0	0	56.67	0	0	0	0	43.33
Data source	55.95	4.26	0	0	95.74	0	0	0	0	0
O_2_ saturation	61.90	67.31	0	21.15	11.54	0	0	0	0	0
BP	23.81	10	0	0	90	0	0	0	0	0
Pulse	28.57	12.50	0	0	87.50	0	0	0	0	0
RR	27.38	13.04	0	0	86.96	0	0	0	0	0
GCS prehospital	8.33	57.14	0	14.29	28.57	0	0	0	0	0
GCS hospital	2.38	50	0	0	50	0	0	0	0	0
Immobilization	54.76	0	2.17	0	23.91	0	0	0	30.43	43.48
Cardiac arrest	3.57	33.33	33.33	0	33.33	0	0	0	0	0
Respiratory airway	4.76	25	0	0	75.00	0	0	0	0	0
Intubation	2.38	50	50	0	0	0	0	0	0	0
Injury date	15.48	0	0	0	100	0	0	0	0	0
Injury cause	4.76	0	0	0	100	0	0	0	0	0
Province of injury occurrence	7.14	0	0	0	66.67	0	0	0	0	33.33
EMS call time	13.10	54.55	18.18	9.09	18.18	0	0	0	0	0
EMS arrival time	11.90	60	20	10	10	0	0	0	0	0
EMS dispatch time	19.05	43.75	12.50	6.25	37.50	0	0	0	0	0
Hospital arrival time	0	0	0	0	0	0	0	0	0	0
Triage admission time	5.95	20	0	0	80	0	0	0	0	0
Inpatient admission	35.71	3.33	0	0	96.67	0	0	0	0	0
Victim position in the traffic accident	36.90	100	0	0	0	0	0	0	0	0
Place of injury event	45.24	2.63	0	0	15.79	0	0	0	81.58	0
Height in falling	9.52	12.50	12.50	0	75	0	0	0	0	0
Comorbidities	19.05	31.25	62.50	0	6.25	0	0	0	0	0
ICU length of stay	0	0	0	0	0	0	0	0	0	0
Activity in event	10.71	0	0	0	100	0	0	0	0	0
Safety device	8.33	14.29	0	0	85.71	0	0	0	0	0

NTRI, National Trauma Registry of Iran; NSCIR-IR, National Spinal Cord Injury Registry of Iran.

**Table 5:** Frequency of Types of Disagreement in Two Registry Centers

**Type of Discrepancy**	**The Number of Variables with Inconsistency**	
	**Sina, Tehran**	**Beheshti, Kashan**
Missing in NTRI	17	19
Missing in NSCIR-IR	4	7
Missing in both NTRI and NSCIR-IR	3	6
Data value conflict	26	35
Typographical errors in NSCIR-IR	0	0
Typographical errors in NTRI	1	0
Different typographical method	3	3
A different method of data entry	2	4
Different detail of data Value	6	3

NTRI, National Trauma Registry of Iran; NSCIR-IR, National Spinal Cord Injury Registry of Iran. Note: There was no common case in the other two centers.

 The frequent conflict in contact information was found in the home telephone number and address; most of the differences appeared trivial and were probably due to data entry errors, i.e., errors in one or two digits of the telephone number. However, the importance of contact information should not be underestimated, as it is essential for patients’ health status follow-up. In some cases, the phone numbers or addresses were completely different, which the expert panel believed was due to different sources used.

 Regarding the occurrence of injuries, including time, cause, location of the incident, the activity of the person at the time of injury, the height of falling, and use of safety equipment, there is not complete consistency between the two registries, mainly due to different data sources and different data entry methods. Prehospital data entry errors were found to be due to the inadequacies of the ambulance forms. Similar to NTRI, the patient or the person accompanying the patient was asked to complete the required sections in the NSCIR-IR registry to fill in missing data in the outpatient form.

 A comparison of data entry in the two registries revealed that patient names, prehospital intubation, ambulance arrival time, length of ICU stay, and admission time were recorded in the same way in both registries.

 The study found numerous data inconsistencies and conflicts in patient vital signs and immobilization, cardiac arrests, and respiratory status in the prehospital data values. However, in some cases, prehospital information was recorded only in the NSCIR-IR; both registries missed important prehospital information because of missing data in the data source. The quality of data in hospitals also varied. Data quality in the level of centers in each registration system, are shown in Table 5.

## Discussion

 NSCIR-IR and NTRI data evaluations revealed similarities in patient names, intubation times, ambulance arrival times, ICU stays, and admission times. Notable differences in the epidemiological data included occupation, date of birth, and contact information. Regarding clinical data, the highest seen diversities were in prehospital vital signs, immobilization, cardiac arrests, and respiratory illnesses. Nevertheless, data quality also varied in the level of centers in each registration system.

 Sonsilphong et al classified and described the various types of data-level conflicts. The concept of different data values in our research is in concordance with Sonsilphong’s definition of data value conflict, which means semantically equivalent data elements with different inputted values.^21^ The concept of different data input in our study matches the concept of data format conflict in Sonsilphong and colleagues’ study, defined as the different format of entered values for semantically equivalent data elements.^21^ Data scaling conflict was another type of conflict used by Sonsilphong et al to refer to different scales or units of measurement for semantically equivalent data items. The present study equally considered this type of conflict. However, no data were found to have the potential for this type of discrepancy. Another observed conflict that was not mentioned by Sonsilphong et al classification was different data details, which was observed only for unstructured data items in the present study.

 A systematic review by Eden et al. examined barriers and factors to HIE and divided them into three general groups: (1) Completeness of data, (2) the internal organization of each system, and (3) the technology, and preliminary goals and benefits of each system.^15^

 In this study, we evaluated only the completeness and structure of the data. Four main identified barriers related to data completeness in this study were:

###  Different Case Finding Rates in the Two Registries

 Case finding involves identifying (i.e. capturing) eligible patients from existing sources using a defined case definition.^22^ As components of the case-finding method, the location of case-finding (emergency department versus specialty department) and the source of case-finding (list of emergency patients in the last 24 hours in the NTRI versus list of emergency patients and frequent visits to the wards and ICU, and registrars’ interactions with residents and ward managers in the NSCIR-IR) differed between the two registries; therefore, the occurrence of differences in case-finding rates or missed cases is inevitable. In addition, it is inevitable that there will be interruptions in case finding because of staff shortages in both registries.^17^ Because coverage in both registries is less than ideal, HIE does not seem to have good prospects unless the two registries are merged and a decision is made to use a standard method with better coverage. It should be noted that although more eligible spine trauma cases are identified in NSCIR-IR, according to the results of this study, neither registry has complete coverage.

 It is well known that completeness of case ascertainment is a critical quality criterion of registries and surveillance studies. In NSCIR-IR, case coverage is usually assessed by comparing registered patients with the patient list derived from the hospital information system using ICD-10 diagnosis codes,^17^ similar to what is done in non-population-based registries based on quantitative assessment methods.^23-25^ However, the present study did not include an assessment of the quality of case ascertainment because assessing the completeness of case finding in NTRI or NSCIR-IR was not the aim of the present study.

###  Missing Data

 The results of the present study show that there are missing values for several variables in both registers. In both registries, there is a large number of missing data due to documentation deficiencies in the outpatient reports. The number of variables with missing values was higher in the NTRI. According to the expert panel, this could be due to the NTRI’s higher dependence on the HIS system, whereas in the NSCIR-IR the registrar is more active in data collection. Therefore, a change in the current data collection process, including data extraction from the NTRI, could affect data quality in the NSCIR-IR. In the eHealth and EHR ecosystem, data sharing between different systems is seen as a means to reduce missing data in information systems.^26^ It is illogical to combine two databases, which would increase the missing data.^27^ According to previous works, differences in the mandatory or optional nature of data elements between different databases and health care information systems are another challenge to electronic clinical data exchange.^27,28^

###  Differences in Data Values (Data Value Conflict)

 Based on studies and technical frameworks, data value conflicts pose a significant challenge to heterogeneous database integration and interoperability.^29^ Although previous work acknowledges the creation of ontologies as a solution for identifying and resolving value conflicts,^30,31^ the challenges of determining the correct data to resolve a conflict have not been addressed. According to this study, it cannot be properly assessed which register is the more accurate one. Improving the quality of data entry should be a priority for both registries.

###  Differences in Detail

 According to the results presented in Table 3, the two registries differed in the details of the entered data for seven common variables, including the vehicle type EMS (air/ground) which in NTRI was separated, but it was not separated in NSCIR-IR; patient immobilization in the prehospital phase in NSCIR-IR, which was recorded as collar or board only; occupation as the occupational category in the trauma registry and the exact occupational title in NSCIR-IR; differences in address and telephone number details; province and city of residence and location of injury. Although this is considered a discrepancy, the difference in detail between the two registries is due to the purpose of the registries rather than a difference in data quality. In each case, the policy committees of the two registries should decide whether it is necessary to standardize other variables and make significant changes due to differences in protocols and registration methods.

## Limitations

 It is important to note that the study had some limitations. Specifically, it only examined the quality and structure of the registries’ data, without a comprehensive evaluation of the barriers that could limit the successful HIE. Additionally, there is no consensus on the acceptable level of difference in data to consider a successful HIE, as this varies depending on the specific goals of each registry and its users.

## Conclusion

 This study showed that although a group of common patients are registered in both registries, in order to perform successful HIE, a wide range of changes are needed, including refinement of the variables, which may not fit the main purpose of the establishment of each registry. Our study serves as a starting point for policymakers and stakeholders to better understand the distinctions between NSCIR-IR and NTRI to make future decisions.
